# Search for Potential VDR/Partner Composite Elements in Regulatory DNA of Genes Associated with Respiratory Infections and Atopic Diseases

**DOI:** 10.3390/ijms27010409

**Published:** 2025-12-30

**Authors:** Alexey V. Popov, Dmitry Yu. Oshchepkov, Vladislav V. Kononchuk, Tatiana S. Kalinina, Ilya S. Valembakhov, Alexander D. Lukin, Elena G. Kondyurina, Vera V. Zelenskaya, Valentin Vavilin

**Affiliations:** 1 Institute of Molecular Biology and Biophysics, Federal Research Center of Fundamental and Translational Medicine, Novosibirsk 630060, Russia; 2 Institute of Cytology and Genetics, Novosibirsk 630090, Russia; 3 Department of Natural Sciences, Novosibirsk State University, Novosibirsk 630090, Russia; 4 Department of Pediatrics, Faculty of Advanced Training and Professional Retraining, Novosibirsk State Medical University, Novosibirsk 630091, Russia

**Keywords:** VDR, NR2C2, PPARG, composite elements, atopy, lung infection

## Abstract

Vitamin D deficiency is associated with the risk of atopic diseases and respiratory infections. The activated vitamin D receptor (VDR) forms a dimer with the retinoid X receptor alpha (RXRA) and binds to VDR/RXRA composite elements (CEs) in enhancers of target genes. However, VDR/RXRA CEs are identified in only 11.5% of cases in ChIP-Seq peaks. Our hypothesis was that VDR could form a VDR-Partner complex with transcription factor for which CEs have not yet been identified. We utilized Web-MCOT to search for novel VDR/Partner CEs in regulatory DNA. The potential formation of the VDR-Partner protein complex was assessed using the AlphaFold machine learning model. Through real-time RT-PCR, we measured the expression of immune system genes in a culture of U937 macrophage-like cells incubated with the active metabolite of vitamin D, calcitriol. We have predicted novel VDR/NR2C2 and VDR/PPARG CEs in the regulatory regions of immune system genes. We found potential synergism of VDR/NR2C2 and VDR/RXRA CEs in relation to the *IRF5* gene, as well as potential synergism of VDR/PPARG and VDR/RXRA CEs for *MAPK13*. Predicting new regulatory relationships through the identification of new potential VDR/Partner CEs may provide insight into the deep mechanisms of vitamin D involvement in the pathogenesis of atopic dermatitis, bronchial asthma, allergic rhinitis, and pulmonary infections.

## 1. Introduction

Vitamin D deficiency is associated with the risk of atopic diseases and respiratory infections [1,2,3]. Vitamin D deficiency is also associated with the severity of COVID-19 [4]. Increasing evidence suggests a link between inadequate vitamin D levels in children and the frequency of respiratory infections, as well as their long-term consequences (such as wheeze) [5]. A compelling link has also been established between vitamin D deficiency and the severity of respiratory infections in infants and children [1,6,7]. Vitamin D levels at birth are associated with the development of respiratory syncytial virus bronchiolitis later in life. Newborns with cord blood 25OHD levels less than 20 ng/mL had an increased risk of developing lower respiratory tract infection in the first year of life compared to those with levels greater than 30 ng/mL [8]. Moreover, in a cohort of newborns hospitalized due to acute lower respiratory tract infections, 25OHD levels were significantly lower at 9 ng/mL compared to the control group at 16 ng/mL [9]. Research indicates that vitamin D supplementation can offer preventive benefits against influenza [3] and COVID-19 [10]. Furthermore, it has been observed that vitamin D can ameliorate symptoms in patients with COVID-19 [11]. Notably, vitamin D deficiency has been associated with asthma severity [1], and vitamin D supplementation may help reduce the severity of asthma and atopic dermatitis [12,13]. The active forms of vitamin D, calcitriol and 1,25-dehydroxyergocalciferol, are produced from cholecalciferol or ergocalciferol, respectively, through a series of hydroxylations at the 25- and 1ɑ-positions. 25-hydroxyvitamin D is synthesized in the liver by the enzyme vitamin D-25-hydroxylase. The subsequent 1ɑ-position hydroxylation occurs in immune system cells through CYP27B1 [14,15]. Cholecalciferol is obtained through dietary sources (primarily fatty fish) or synthesized in the skin from 7-dehydrocholesterol. Conversely, ergocalciferol is only available through food sources (mostly mushrooms) [16]. VDR is expressed in nearly all immune cells, including macrophages [8,17].

Macrophages play a crucial role in the etiopathogenesis of respiratory infections and atopy [18,19]. These immune response cells are involved not only in innate but also adaptive immunity. The immunomodulatory effects of vitamin D on macrophages are best explained by the binding of VDR to vitamin D response element (VDRE), as well as the interaction of VDR with inflammasome and IκB kinase β proteins [20,21]. Vitamin D increases the expression of mitogen-activated protein kinase phosphatase-1 (*MKP-1*) in monocytes and macrophages [22], leading to reduced TNFa and IL6 production and inhibition of p38 phosphorylation [23]. VDREs have been identified in the *MKP-1* promoter region in mice and humans. MKP-1 deficiency results in overproduction of numerous factors, including proinflammatory cytokines TNFa, IL6, IL1B [24,25,26] anti-inflammatory cytokine IL10 [27,28,29], MIP-1α, MIP-1β, MCP-1 and CXCL2 [28,30], iNOS [31,32] and cyclooxygenase 2 [33]. Vitamin D can enhance *TNFa* expression by activating VDR, which binds to the VDRE in the *TNFa* promoter [34]. 1,25-Dihydroxyvitamin D bound to VDR directly induces transcription of cathelicidin, human beta-defensin 2, and *IL1B* via the VDRE present in the proximal promoters of the corresponding genes [35,36,37].

In our work, we examined the putative genomic interaction of VDR with novel potential cis-regulatory elements. Activated VDR forms a heterodimeric complex with RXRA and binds to hexameric A/GGG/TTC/GA motifs separated by a 3-nucleotide spacer, which are called DR3-responsive elements [38] or CEs. In ChIP-Seq peaks, CEs to the VDR-RXRA heterodimer are identified in only 11.5% of cases [39]. This may be due to the fact that studies on the possibility of forming overlapping VDR/RXRA motifs have not been previously conducted. Also, no analysis has been performed for the presence of other VDR/Partner CEs. The only tool for searching for overlapping motifs and motifs with spacers in a single ChIP-Seq is the Motifs Co-Occurrence Tool (MCOT). For a given “Anchor” motif and a set of input peaks, Web-MCOT checks the significance of CEs with various partner motifs from libraries obtained from the entire genome [40]. Also, relatively recently, the ability to model protein–protein interactions based on the AlphaFold machine learning model [41] has become possible, which provides additional arguments in favor of the functional activity of VDR complexes with unknown partners. The aim of this work was to test our hypothesis that VDR can form complexes with transcription factors for which CEs have not yet been found and to identify potential partners for VDR and their CEs that may be involved in the regulation of genes associated with atopy and pulmonary infections.

## 2. Results

### 2.1. Bioinformatics Analysis Results

According to MCOT computations, the Top 10 motifs that have the potential to create CEs with VDR encompass the already established RXRA, alongside PPARG, PPARA, and NR2C2 (refer to Table 1).

**Figure 1:**
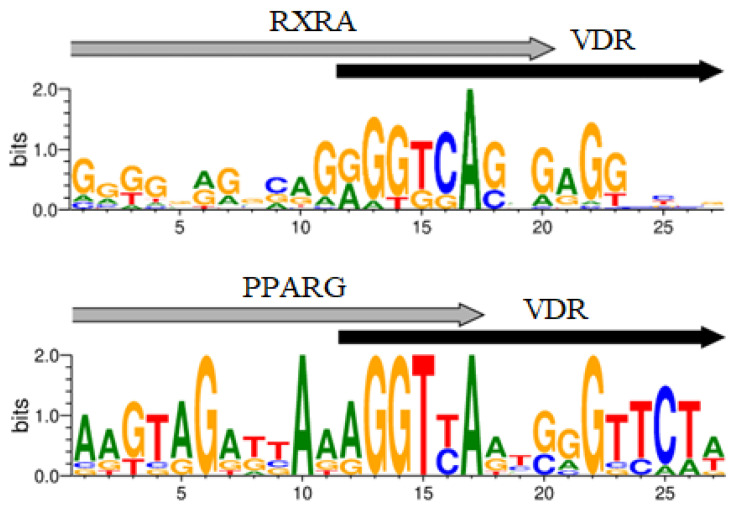

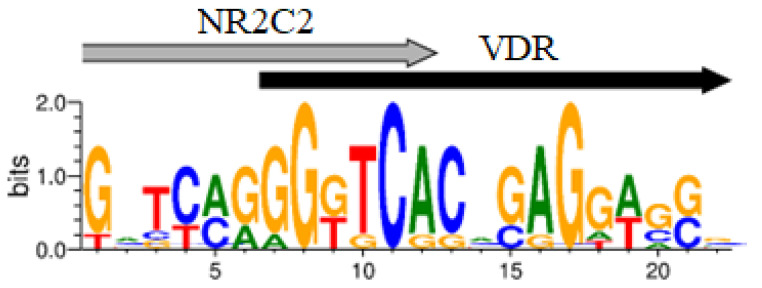
Logos of the VDR/RXRA, VDR/PPARG, VDR/NR2C2 novel potential CEs.

The BioGRID database, which compiles information on protein–protein interactions, contains data on the interaction of VDR transcription factor (TF) with RXRA and PPARG. A protein complex formation was demonstrated for VDR-PPARG in only one study to our knowledge [42]. It was observed that NR2C2 and PPARG recognize similar DNA sequences [43], hence NR2C2 TF could also be considered a partner of VDR. The consensus sequence for PPARG conforms to the classic version: vWbbRGGbSARAGGKSR (with a *p*-value of 2.61 × 10^−10^, E-value of 5.40 × 10^−07^, and q-value of 1.07 × 10^−06^), similar to PPARA (*p*-value = 4.26 × 10^−09^, E-value = 8.83 × 10^−06^, q-value = 8.74 × 10^−06^). The CEs for VDR-PPARA and VDR-PPARG in the target genes we identified showed substantial overlap in almost all cases. Therefore, the presence of PPARA in the MCOT output may be an artifact. Additionally, the top 10 motifs in the MCOT output included TFs like ATF2, MITF, TFE3, SPI1, USF1, and REL. However, none of these were listed as VDR partners in the BioGRID database. Consequently, the most probable VDR partners are the well-known TF RXRA (*p*-value = 6.0 × 10^−30^), as well as PPARG (*p*-value = 2.1 × 10^−22^) and NR2C2 (*p*-value = 9.1 × 10^−34^). The logos of potential VDR/Partner CEs are depicted in Figure 1.

To validate this prediction further, we built the VDR-NR2C2 protein complex using the AlphaFold program (Supplementary Figure S1).

Next, we counted all genes (98) whose regulatory DNA contained predicted CEs for the VDR-RXRA, VDR-PPARG, and VDR-NR2C2 complexes, according to the results of MCOT calculations on the VDR ChIP-Seq data for the THP-1 monocytic cell line culture (Table S1). 72 genes contained CEs in the VDR/RXRA regulatory regions: 15 genes contained VDR/PPARG; 16 genes contained VDR/NR2C2; 27 genes contained all three CEs variants; and 14 genes included neither VDR/NR2C2 nor VDR/PPARG. We also identified 26 genes that did not have VDR/RXRA in their enhancers but did contain VDR/NR2C2 and/or VDR/PPARG.

Note: Table S1 consists of information about occurrence or absence of potential CEs in enhancers of all 98 genes.

Among the ChEA 2022 genes we identified, the analysis revealed that, in addition to VDR, PPARG was among the top 10 regulators (see Table 2).

The genes with the highest search score in the aspect of bronchopulmonary infections and atopy from Table S1 in the Malacards search results (Table 3) were: *NOD2*, *LGALS9*, *MAPK13*, *PDCD1LG2*, *NFKBIA*, *CD14*, *IRF5*.

The NOD2 receptor (Nucleotide Binding Oligomerization Domain Containing 2) is a cytosolic protein involved in inflammatory processes associated with NFkB activation. NOD2 enhances NFkB transactivation in transfected cells [44]. The *LGALS9* gene encodes galectin-9, a 36 kDa beta-galactoside lectin protein. Galectin-9 is an activator of NFkB, as Galectin-9 deficiency in dengue virus-infected dendritic cells suppressed this transcription factor [45]. MAPK13, a mitogen-activated protein kinase, is involved in the synthesis of IL6 [46]. PDCD1LG2 (PD-L2) (Programmed Cell Death 1 Ligand 2) is a transmembrane protein and a ligand for PD1. It prevents T-lymphocyte activation. PDCD1LG2 has an immunomodulatory effect on IL-10 [47,48]. It increases IFNg production. NFKBIA (NFkB Inhibitor Alpha) interacts with the p65, p50, and p52 subunits of NFkB [49,50,51]. CD14 is a receptor involved in regulating the production of IL10, IL6, IL8, and TNFa [52,53,54,55]. IRF5 is a TF involved in the regulation of IFNa, INFb, and IL12 [56].

### 2.2. Experimental Study Results on the Impact of Vitamin D on the Expression of Target Genes in U937 Cell Culture. Comparison of Experimental Findings in U937 Cells with Bioinformatics Analysis in THP-1 Cells

The next step was to confirm vitamin D-mediated regulation of the genes for which new CEs were identified in this study.

**Figure 2:**
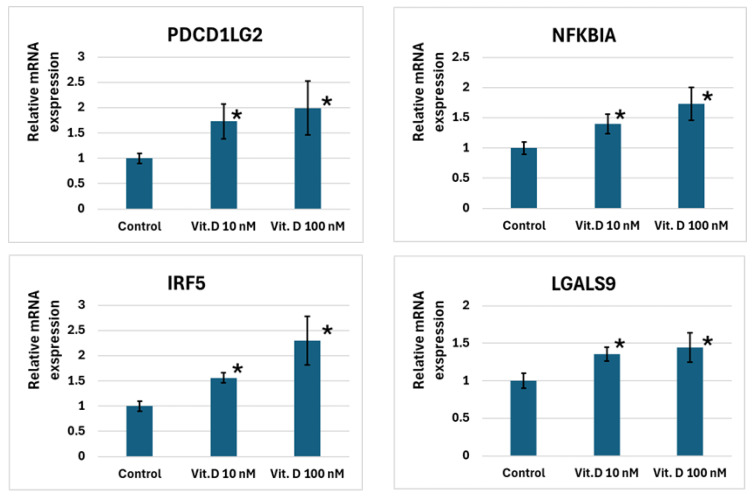

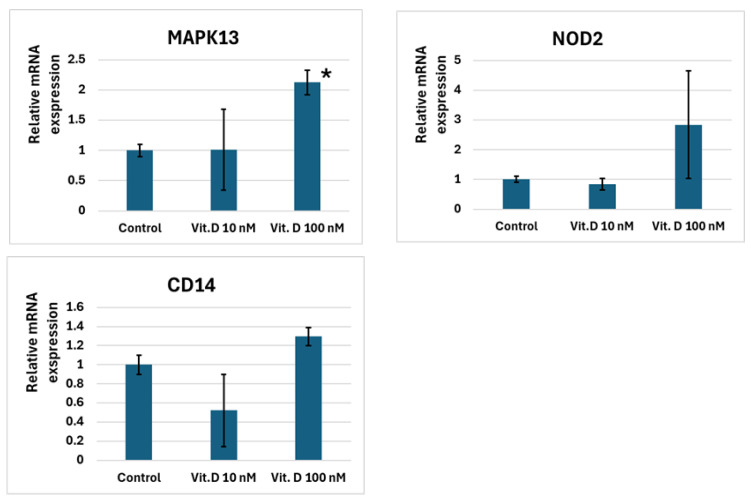
Changes in immune system gene expression levels in U937 cells exposed to 10 and 100 nM calcitriol. Note: *—*p* < 0.05, where *p* is the significance level of differences with the control.

In U937 cells, 10 nM and 100 nM calcitriol increased the expression levels of *PDCD1LG2*, *NFKBIA*, *IRF5*, and *LGALS9*. *MAPK13* expression increased only at a calcitriol concentration of 100 nM. *NOD2* and *CD14* did not respond to calcitriol (Figure 2).

Among the genes that exhibited increased expression in response to calcitriol, the most pronounced change was observed for *IRF5* (refer to Figure 2). A potential VDR/NR2C2 CE was detected in the enhancer region of the gene about ~−2.3 kb from the transcription start site (TSS), along with another potential VDR/RXRA CE at about ~−25.1 kb from the TSS (see Table 4). The impact of 100 nM calcitriol on *MAPK13* expression approximated that of *IRF5*. In the gene’s promoter region, we predicted two VDR/RXRA CEs about ~−0.3 kb from the TSS, together with another VDR/PPARG CE roughly ~+168.5 kb from the TSS.

We also noted an increase in *LGALS9* expression. A potential VDR/Partner CE (with the partner being either RXRA or PPARG) was identified in the gene’s promoter about ~+1.2 kb from the TSS, and another potential VDR/NR2C2 CE approximately ~−297.2 kb from the TSS.

*IRF5* and *MAPK13* showed the most pronounced expression changes compared to *PDCD1LG2* and *NFKBIA*. Regarding the *PDCD1LG2* gene, we cannot consider the synergy of CEs since we only spotted one potential VDR/Partner CE (where the partner can be RXRA or PPARG), situated at a distance of ~−73 kb from the TSS. Thus, in this case, we only consider the competitive binding of the respective TFs. Concerning *NFKBIA*, we also discovered only one potential VDR/Partner CE (with the partner possibly being RXRA, PPARG, or NR2C2) around ~−1.3 kb from the TSS, leading us to consider only competitive binding here as well.

## 3. Discussion

### 3.1. Physiological Role of Vitamin D

Vitamin D plays a crucial role in human physiology and pathology. Apart from regulating calcium/phosphate metabolism, vitamin D is implicated in safeguarding against cancer chemoprevention, enhancing the cardiovascular system, detoxifying xenobiotics, shielding against neurodegenerative diseases, immunoregulation, and providing antimicrobial protection [57,58].

### 3.2. The Role of Vitamin D in the Differentiation of Macrophages and Macrophage-like Cells

Macrophages are among the most critical cells in the immune system. Vitamin D increases Tim-3 expression, leading to M2 polarization [59]. It has also been demonstrated that Vitamin D enhances *IRF5* expression in THP-1 cells, indicating the formation of M1 quasi-macrophages [39]. Therefore, Vitamin D may play a role at all stages of the inflammatory process, helping to maintain homeostasis and regulate the number of macrophages with the desired phenotype.

### 3.3. Role of the Studied Vitamin D Target Genes in the Immune Response

Galectin-9 has an immunomodulatory effect on *IL17* expression [60,61,62]. In a polymicrobial sepsis model, galectin-9 improved animal survival [61]. It reduced levels of IL6, IL10, HMGB1, and increased levels of IL15 and IL17 in plasma and spleen. Galectin-9 increased the count of natural killer T cells (NKT cells) and PDCA-1+ CD11c+ macrophages. Galectin-9 has demonstrated efficacy in a Dermatophagoides farinae allergen-induced asthma model by suppressing the expression of *IL5*, *IL13*, *CCL11*, *CCL17*, reducing eosinophilia, and pulmonary hyperresponsiveness [63]. Incubation of peripheral blood mononuclear cells with galectin-9 resulted in increased production of IL6 and IL10 [64]. Galectin-9 induced the secretion of TNFa, IL1B, IFNg, IL10, IL4, IL13 from peripheral blood mononuclear cells [65]. Galectin-9-induced dendritic cells stimulated the secretion of IFNg, IL10, TNFa, and IL2 from CD4+ T cells [66]. Furthermore, galectin-9 was found to suppress *IFNg* expression, indicating its immunomodulatory effects on this cytokine [67]. Galectin-9 also inhibited the formation of IgE-antigen complexes, thus preventing mast cell degranulation [68]. In an RSV infection model, galectin-9 increased the number of T regulatory cells, *IL10* expression, and suppressed the Th17 response [69]. Galectin-9 binds to ACE2, inhibiting SARS-CoV-2 entry, reducing cell infection in vitro, and improving animal survival [70].

Polymorphic variants of *CD14* are linked to the risk of complications in atopy and respiratory viral infections [53,71,72].

Polymorphic variants of *NOD2* are associated with bronchial asthma [73].

*MAPK13* demonstrates pro-inflammatory effects in a model of post-viral lung disease [74] but has not been thoroughly studied in asthma models.

IRF5 activates macrophages in response to the virus and promotes the M1 phenotype [75,76]. Overactivation of *IRF5* can trigger a cytokine storm in COVID-19 [77]. In an atopic asthma model, IRF5 reduces lung hyperresponsiveness, mucus production, and IL13 [78].

Polymorphic variants of *PDCD1LG2* are associated with allergic rhinitis [79].

*NFKBIA* is one of the most critical genes in the etiopathogenesis of bronchopulmonary diseases. Polymorphic variants in the *NFKBIA* promoter are linked to an increased risk of hospitalization in severe RSV bronchiolitis and pulmonary hyperresponsiveness among children with respiratory viral infections identified within the first 12 months of life [80,81]. Polymorphic variants of *NFKBIA* are also associated with the risk of COVID-19 infection [82].

### 3.4. Putative Synergism and Predicted Competing Effects of New Potential CEs on Immune Response Genes: Involvement of NR2C2 and PPARG in Inflammation

In our study, we observed that the combined presence of predicted VDR/RXRA CEs with VDR/NR2C2 or VDR/PPARG could potentially have an additive impact on *IRF5* and *MAPK13*, respectively, in U937 culture. It was noted that the alteration in *LGALS9* expression was not as striking as observed for *IRF5* and *MAPK13*. This discrepancy might be attributed to the likely differences in chromosomal landscapes between U937 and THP-1 cells (these lines have different origin), potentially influenced by deletions, point mutations, and heterochromatin distribution. Moreover, it is worth considering that one of the dimers could potentially bind to the negative VDRE, or not bind at all, in the case of competing binding by other transcription factors that might impair enhanced expression. Within the regulatory regions of the *PDCD1LG2* and *NFKBIA* genes, we predicted the presence of a CE supposing competing binding among VDR partners. Consequently, the anticipated effect might not be as robust as noted for *IRF5* and *MAPK13*. Bioinformatics analysis illustrated equal possibilities of VDR-RXRA, VDR-PPARG, and VDR-NR2C2 complex formations. Nevertheless, given the notably higher expression of RXRA in comparison to PPARG and NR2C2 in cell cultures, it seems plausible to suggest that, in scenarios involving potential competing binding, RXRA would likely displace PPARG and NR2C2 from the complexes. We theorize that in instances of RXRA deficiency, VDR could potentially form a dimer with PPARG or NR2C2, thereby ensuring the functioning of VDR both in cases where potential competing influence of TF is present and in scenarios where potential synergism was detected. PPARG is known to have an immunomodulatory effect [83,84]. Our study indicated that PPARG potentially stimulates the increased expression of *MAPK13*, hinting at its immunostimulatory influence. Strategic regulation of PPARG expression might enable a response to pathogens and help prevent respiratory syncytial virus infections, which are often associated with atopy [85]. On the other hand, NR2C2 is recognized for its proinflammatory effects during bacterial infections as it augments NFkB expression, thereby fostering the production of IL1B and IL6 in macrophages [86]. We predicted that NR2C2 could potentially bind to the *IRF5* promoter, possibly leading to the formation of M1 quasi-macrophages. It is important to note that M1 macrophages in a bronchial asthma model exhibit an anti-inflammatory effect, while showing a proinflammatory effect during bacterial or viral infections, suggesting possible immunomodulatory effects of *IRF5* and NR2C2. In our study, the expression levels of *NOD2* and *CD14* remained unchanged. The ChIP-Seq of VDR conducted on THP-1 cell culture could likely not be directly applicable for the study of *NOD2* and *CD14* expression in U937 cells due to the aforementioned limitations. Although *CD14* did not respond to calcitriol in our experiment, in THP-1 culture this gene is one of the leaders in terms of increased expression [39]. Consequently, we posit that VDR-NR2C2 complexes may potentially contribute to an increased production of this receptor in THP-1 cells.

## 4. Materials and Methods

### 4.1. Bioinformatics Analysis Methods

To search for composite elements in the regulatory regions of genes, we utilized the MCOT program (https://webmcot.sysbio.cytogen.ru/, Novosibirsk, Russia) [40] and ChIP-Seq VDR data from the THP-1 monocytic cell line culture [39,87] obtained from the CistromeDB database (http://cistrome.org/db/, accessed on 11 November 2025, Shanghai, China, Boston, MA, USA) [88]. The VDR weight matrix derived de novo from this data, along with the ChIP-Seq peak sequences, were input into the MCOT following the program’s user manual. The resulting list of potential VDR partners was cross-referenced with the THP-1 RNA-Seq data [39] to identify expressed TFs in THP-1. In instances where the binding of a potential VDR partner to VDRE lacked literature evidence, the Enrichr database (ChEA 2022, New York, NY, USA) [89,90,91] was consulted to investigate the impact of such TFs on the expression of genes directly controlled by vitamin D. VDR-partner protein complex was built using AlphaFold 3 (London, UK) and visualized in the ChimeraX 1.5 (San Francisco, CA, USA) software package [92]. Genes harboring predicted VDR/Partner CEs in their regulatory DNA, and related to the regulation of cytokine production or linked to atopic or viral diseases (such as asthma, atopic dermatitis, allergic rhinitis, and viral lung infections), were selected for further analysis. Subsequently, an experimental investigation into the influence of vitamin D on the expression of vitamin D target genes was conducted (refer to Section 3.2). The potential effectiveness of the new putative CEs was evaluated based on the Haussler model [57], which posits that changes in expression levels are directly correlated to the number of VDR/Partner CEs in the gene’s regulatory regions.

### 4.2. Materials and Methods of the Experimental Study

#### 4.2.1. Cell Culturing

The U937 lymphoma cell line was procured from the Russian Collection of Cell Cultures (St. Petersburg, Russia). The U937 cell line, in contrast to THP-1, originates from tissues (more differentiated) and is derived from pleural effusion, making it a suitable choice for studying atopic asthma and respiratory infections [93]. When choosing a cell culture, we also made sure that VDR, RXRA, PPARG and NR2C2 are actually expressed in U937 [94]. U937 cells were cultured in RPMI-1640 medium supplemented with 10% fetal calf serum and 2 mM L-alanyl-L-glutamine. The cells were maintained in a 5% CO_2_ incubator at 37 °C.

To induce the macrophage phenotype, cells were treated with 12.5 nM phorbol 12-myristate 13-acetate (PMA, Cayman Chemical, Ann Arbor, MI, USA) for 48 h. Subsequently, the medium was aspirated, and cells were washed with phosphate-buffered saline before being incubated in PMA-free medium for an additional 18 h. The resulting macrophage cells were exposed to a 10 and 100 nM solution of the active form of vitamin D3-calcitriol in DMSO, along with a 0.1% (*v*/*v*) DMSO control, for 24 h. Due to 77.5% of genes in THP-1 cells respond only 24 h after stimulation, we selected this time point for U937 [39]. All experimental conditions were performed in triplicate (three independent biological replicates).

#### 4.2.2. Total RNA Isolation

Total RNA was extracted from the cell culture to quantify the mRNA levels using TRIzol Reagent (Thermo FS, Waltham, MA, USA) following the manufacturer’s instructions. The quality of isolated RNA was evaluated through horizontal electrophoresis, and the RNA concentration was measured using a Nano Photometer P-360 spectrophotometer (IMPLEN, Munich, Germany) at 230, 260, and 280 nm wavelengths. A 260/280 ratio of 1.8–2.0 and a 260/230 ratio of 1.8 were deemed satisfactory.

#### 4.2.3. Reverse Transcription

To generate cDNA from the RNA template, the RT M-MuLV–RH kit (BioLabMix, Novosibirsk, Russia) was employed as per the manufacturer’s guidelines.

#### 4.2.4. Quantitative PCR with Real-Time Detection

To assess gene expression levels, real-time RT-PCR was conducted utilizing the BioMaster HS-qPCR SYBR Blue (2×) kit on a CFX96Touch thermal cycler (BioRad, Hercules, CA, USA). GAPDH and 18S were used as reference genes, as they are widely employed and have been previously demonstrated to be suitable stable normalization genes for U937 cells [95,96]. The primer sequences employed in the study are detailed in Table 5. The concentration of all primer pairs in the reaction mix was 300 nM. Each PCR reaction was performed in a 20 μL volume, using 0.3 μL of cDNA. Each sample was analyzed in triplicate (technical replicates).

The reaction protocol involved an initial preheating at 95 °C for 5 min, followed by 40 cycles comprising denaturation at 95 °C for 15 s, annealing at 60 °C for 20 s, and elongation with fluorescence data collection at 72 °C for 30 s. Melting curves were generated to confirm PCR specificity. The relative gene expression levels were determined using the threshold cycle values Ct following the 2−∆∆Ct method. Prior to ΔCt calculation, the geometric mean of Ct values for reference genes was determined. Additionally, electrophoresis on a 3% agarose gel was conducted to verify the quality of the PCR products.

#### 4.2.5. Statistical Data Processing

Data analysis was carried out using MS Office. The results are presented as the mean values. Student’s *t*-test was applied to each gene to perform pairwise comparisons and assess variations in gene expression levels between experimental group and control group. Results with *p* < 0.05 were deemed statistically significant.

## 5. Conclusions

In our study, we investigated the scientific hypothesis regarding the regulation of immune system genes by vitamin D through novel potentially CEs. While the list of genes regulated by VDR has notably expanded over the past decade, it appears to be still incomplete. Investigating the impact of novel potential CEs on gene expression can provide insights into the underlying mechanisms of vitamin D in the pathogenesis of diseases like atopic dermatitis, bronchial asthma, allergic rhinitis and pulmonary infections.

## Figures and Tables

**Table 1:** VDR partner motifs derived from MCOT output.

Motif Name	Full Overlap, *p*-Value	Partial Overlap, *p*-Value	Overlap, *p*-Value	Spacer, *p*-Value	Any, *p*-Value
PPARG	1	2.1 × 10^−22^	2.1 × 10^−22^	1	1
RXRA	6.3 × 10^−29^	2.3 × 10^−15^	6 × 10^−30^	1	2.6 × 10^−07^
PPARA	1	1.3 × 10^−22^	1.3 × 10^−22^	1	5.9 × 10^−06^
NR2C2	1.4 × 10^−11^	6.5 × 10^−25^	9.1 × 10^−34^	1	5.5 × 10^−06^

**Table 2:** ChIP-Seq top 10 regulators of 98 vitamin D target genes.

№	Name	*p*-Value	Adjusted *p*-Value	Odds Ratio	Combined Score
1	VDR 24763502 ChIP-Seq THP-1 Human	6.358 × 10^−45^	4.508 × 10^−42^	24.72	2515.98
2	VDR 24787735 ChIP-Seq THP-1 Human	7.968 × 10^−42^	2.825 × 10^−39^	27.65	2616.57
3	VDR 23401126 ChIP-Seq LCL-AND-THP1 Human	8.539 × 10^−22^	2.018 × 10^−19^	28.00	1358.21
4	IRF8 27001747 Chip-Seq BMDM Mouse	8.847 × 10^−9^	0.000001568	4.51	83.72
5	VDR 21846776 ChIP-Seq THP-1 Human	1.313 × 10^−8^	0.000001862	6.48	117.67
6	PPARG 20176806 ChIP-Seq 3T3-L1 Mouse	3.075 × 10^−8^	0.000003574	4.47	77.40
7	BCL6 25482012 ChIP-Seq CML-JURL-MK1 Human	3.529 × 10^−8^	0.000003574	4.19	71.91
8	ELK3 25401928 ChIP-Seq HUVEC Human	9.817 × 10^−8^	0.000008700	3.96	63.91
9	GATA1 19941827 ChIP-Seq MEL86 Mouse	2.158 × 10^−7^	0.00001700	3.99	61.29
10	LMO2 20887958 ChIP-Seq HPC-7 Mouse	3.020 × 10^−7^	0.00002141	4.16	62.44

**Table 3:** Top 7 vitamin D target genes involved in the etiopathogenesis of respiratory infections and atopy.

№	Gene	MCID	Disorder	MIFTS	Search Score
1	*CD14*	IGR001	Ige Responsiveness, Atopic	42.47	15.22
2	*NOD2*	DRM053	Dermatitis, Atopic	61.18	9.83
3	*NFKBIA*	AST005	Asthma	79.86	7.92
4	*PDCD1LG2*	TBR010	Tuberculosis	59.93	6.74
5	*IRF5*	VRL011	Viral Infectious Disease	44.32	3.30
6	*MAPK13*	LNG099	Lung Disease	55.77	2.50
7	*LGALS9*	VRL011	Viral Infectious Disease	44.32	1.22

Note: MCID—MalaCards database identifier for this disorder. MIFTS—MalaCards InformaTion Score. MIFTS defines the richness of information in each card. Search score: Gene-disease association scores, computed as weighted sums of individual scores derived from OMIM, ClinVar, Orphanet, SwissProt/Humsavar, GeneTests, DISEASES, Novoseek and GeneCards.

**Table 4:** Predicted CEs in the enhancers of genes *IRF5*, *MAPK13*, *LGALS9*, *PDCD1LG2*, *NFKBIA*, *NOD2* and *CD14* were identified in THP-1 cells.

Potential CE	VDR Motif	Partner Motif	Distance Between Potential CE and TSS	Gene	GeneHancer Identifier/ GH Type
VDR/RXRA	aggtcacagaggaaag	gcctggagctgaggtcacag	−25,133	*IRF5*	GH07J128909/ Promoter
VDR/NR2C2	aggtcaacaggtctga	gctctgaggtca	−2296	*IRF5*	GH07J128932/ Enhancer
VDR/RXRA	gggtcactcaggtcat	ggaggaggagagggtcactc	−251	*MAPK13*	GH06J036126/Promoter
VDR/RXRA	gggtcactcaggtcat	gggaggggaggaggagaggg	−251	*MAPK13*	GH06J036126/Promoter
VDR/PPARG	aggtcaaagggtttac	caccaggtcaaagggtt	168,537	*MAPK13*	GH06J036297/ Enhancer
VDR/NR2C2	aggtgacccgggcctt	aggcccgggtca	−297,160	*LGALS9*	GH17J027331/ Promoter
VDR/RXRA	aggtcactggggctgg	ggaggagggaaaggtcactg	1242	*LGALS9*	GH17J027630/ Promoter
VDR/PPARG	aggtcactggggctgg	ggaggagggaaaggtca	1242	*LGALS9*	GH17J027630/ Promoter
VDR/RXRA	gggtcagcccggccat	ggtggggagaagggtcagcc	−72,794	*PDCD1LG2*	GH09J005435/ Promoter
VDR/PPARG	gggtcagcccggccat	ggtggggagaagggtca	−72,794	*PDCD1LG2*	GH09J005435/ Promoter
VDR/RXRA	agttcacggagttcac	cacagagtcagagttcacgg	−1324	*NFKBIA*	GH14J035396/Promoter
VDR/PPARG	agttcacggagttcac	cacagagtcagagttca	−1324	*NFKBIA*	GH14J035396/Promoter
VDR/NR2C2	agttcacggagttcac	agtcagagttca	−1324	*NFKBIA*	GH14J035396/Promoter
VDR/RXRA	aggtcactggtgacag	tgagcaatgggaggtcactg	2225	*NOD2*	GH16J050695/ Promoter
VDR/NR2C2	gggtcaccggggtgcc	gcctcggggtca	16,038	*NOD2*	GH16J050710/ Enhancer
VDR/NR2C2	ggttcaagcagttctc	cttccgggttca	−33,724	*CD14*	GH05J140663/ Promoter
VDR/NR2C2	acttcagggaggtcga	aggtcgaggtcg	−15,685	*CD14*	GH05J140646/Promoter
VDR/NR2C2	gggacgccagggtcac	aggtcgaggtcg	−15,705	*CD14*	GH05J140646/Promoter
VDR/NR2C2	gggacgccagggtcac	acgccagggtca	−15,705	*CD14*	GH05J140646/Promoter
VDR/RXRA	ggttcacagaggaggg	gaggtgatcagggttcacag	−1500	*CD14*	GH05J140635/ Enhancer
VDR/NR2C2	ggttcacagaggaggg	gatcagggttca	−1500	*CD14*	GH05J140635/ Enhancer
VDR/PPARG	aggttaatgggttcta	aagtagattaaaggtta	24,519	*CD14*	GH05J140609/ Promoter
VDR/NR2C2	gggttggagggtgcag	gcttagggttgg	36,255	*CD14*	GH05J140596/ Promoter
VDR/NR2C2	gggtcagggaggtcac	gtccaagggtca	309,648	*CD14*	GH05J140322/ Enhancer
VDR/PPARG	gggtcagggaggtcac	acctggtccaagggtca	309,648	*CD14*	GH05J140322/ Enhancer

Note: Table 4 partially duplicates Table S1 and consists detailed information about potential CEs in enhancers of *IRF5*, *MAPK13*, *LGALS9*, *PDCD1LG2*, *NFKBIA*, *NOD2*, *CD14* genes.

**Table 5:** Primer sequences for real-time RT-PCR.

Gene	Primer	Sequences
*18S*	Forward	5′-CGGCTACCACATCCAAGGAA-3′
	Reverse	5′-GCTGGAATTACCGCGGCT-3′
*GAPDH*	Forward	5′-ACAACTTTGGTATCGTGGAAGGAC-3′
	Reverse	5′-CAGGGATGATGTTCTGGAGAGC-3′
*NFKBIA*	Forward	5′-ACTCGTTCCTGCACTTGGC-3′
	Reverse	5′-CGAAAGTCTCGGAGCTCAG-3′
*IRF5*	Forward	5′-ATGAGCTCATCCTGTTCCAAAA-3′
	Reverse	5′-ATAGCTCCCCTGAGAACATC-3′
*LGALS9*	Forward	5′-GCTGTGAACTTTCAGACTGG-3′
	Reverse	5′- ACCATCACCTTGAAATCTGAGC-3′
*NOD2*	Forward	5′-TCATCTGGCTCATCCGGAG-3′
	Reverse	5′-AAATACAGAGCCTTGCAGACAC-3′
*MAPK13*	Forward	5′-GGGATGGAGTTCAGTGAGGA-3′
	Reverse	5′-TCACCACGTAGCCAGTCATC-3′
*PDCD1LG2*	Forward	5′-CAGATAGCAGCTTTATTCACAGT-3′
	Reverse	5′-TTGAGGTATGTGGAACGAGG-3′
*CD14*	Forward	5′-GACCATGGAGCGCGCGT-3′
	Reverse	5′-TGGAAGGCTTCGGACCAG-3′

## Data Availability

The data presented in this study are unavailable due to privacy.

## Supplementary Materials

The following supporting information can be downloaded at: https://www.mdpi.com/article/10.3390/ijms27010409/s1.
