# Supplementary material for: Search for Potential VDR/Partner Composite Elements in Regulatory DNA of Genes Associated with Respiratory Infections and Atopic Diseases
Source: Int J Mol Sci. 2025 Dec 30;27(1):409. doi: 10.3390/ijms27010409 (PMC12786173; doi:10.3390/ijms27010409)
Supplement: Supplementary file 1 [file ijms-27-00409-s001.zip › Supplementary Figure S1.pdf]

(a)

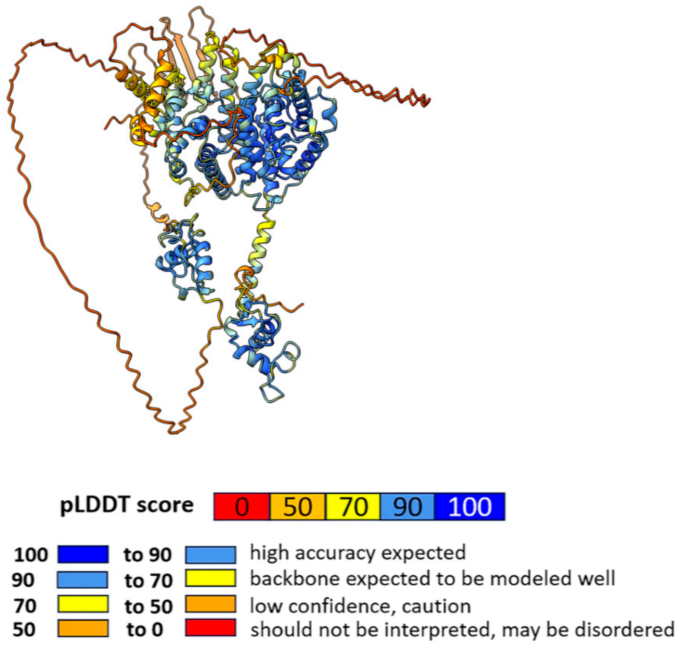

(b)

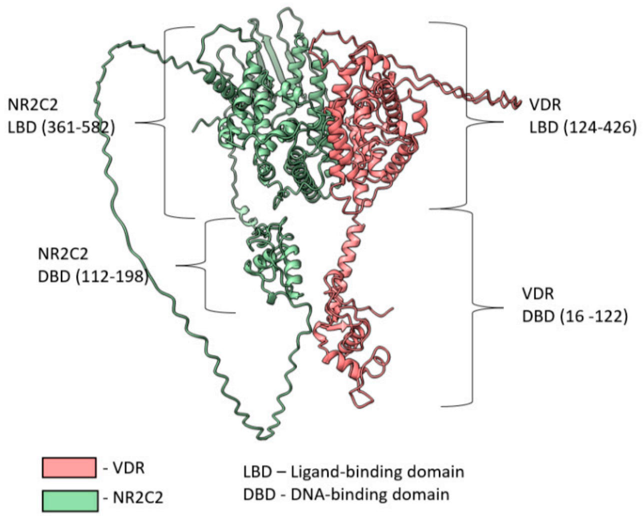

Prediction of the VDR-NR2C2 dimer structure in AlphaFold: (a)VDR-NR2C2 pLDDT, (b) Domain structure of VDR-NR2C2
